# Enhancing home-based physical activity for neurodivergent children: adapting the *InPACT at Home* program with AI and universal design

**DOI:** 10.3389/fphys.2024.1496114

**Published:** 2025-01-07

**Authors:** Tania Sapre, Haylie L. Miller, Anna Schwartz, Leah R. Ketcheson, Alanna Price, Kerry Winkelseth, Jeanne M. Barcelona, Ronald F. Zernicke, Rebecca E. Hasson

**Affiliations:** ^1^ School of Kinesiology, University of Michigan, Ann Arbor, MI, United States; ^2^ College of Education, Wayne State University, Detroit, MI, United States; ^3^ Detroit Public School Community District, Detroit, MI, United States; ^4^ Department of Orthopaedic Surgery, School of Medicine, University of Michigan, Ann Arbor, MI, United States

**Keywords:** generative AI, universal design for learning (UDL), adolescent and youth, exercise, implementation science

## Abstract

**Purpose:**

While it is common practice for schools across the United States to include neurodivergent children in physical education classes, many programs outside of school—such as those at home or in the community—are not effectively tailored to meet their support needs. This gap contributes to lower levels of physical activity among neurodivergent children. Our objective was to address this issue by systematically adapting the *InPACT (Interrupting Prolonged sitting with ACTivity) at Home* program to enable neurodivergent children to safely engage in physical activity at home.

**Methods:**

The rapid-cycle research adaptation process involved several key steps: (1) sorting and grouping video content based on different types of skills and exercises (*problem exploration*); (2) assembling an expert team to guide the development of the instructions (*knowledge exploration*); and (3) using generative artificial intelligence (AI) to create concise instructions and cue words for each skill/exercise (*solution development*). These outputs were then fine-tuned by the expert team. The refinements were guided by the Universal Design for Learning (UDL) principle of “Representation,” which acknowledges that learners perceive and understand information in diverse ways.

**Results:**

From the 132 *InPACT at Home* videos, over 500 activities were identified and categorized into main skill groups: jumping, core, lateral, sport, upper body, lower body, and compound movements. Expert meetings emphasized the importance of the “Three C’s”—consistency, conciseness, and clarity—in crafting instructions, along with the use of simple, elementary sight words. AI was employed to generate and refine prompts like “Provide simplified step-by-step instructions for a jumping jack, suitable for a neurodivergent child” and “Condense the step-by-step instructions for a jumping jack, suitable for a neurodivergent child”.

**Discussion:**

The adaptation of the existing *InPACT at Home* program was guided by dissemination and implementation science frameworks, aiming to increase equitable access to structured youth physical activity opportunities for neurodivergent children. By incorporating AI and UDL principles, we aim to further enhance the program’s accessibility. Our next steps include evaluating the effectiveness of our program adaptations in encouraging participation in the *InPACT at Home* program and subsequently increasing physical activity levels among neurodivergent children.

## Introduction

### The role of physical activity in child development

Physical activity plays a critical role in shaping children’s physical health, social development, and cognitive abilities. For neurotypical children, participation in physical activity is associated with improved physical health, higher self-esteem, and the development of a strong social identity through sports (Logan et al., 2019; Piercy and Troiano, 2018). Similarly, children with intellectual and developmental disabilities experience comparable benefits. For instance, neurodivergent children (e.g., those diagnosed with autism, developmental delay, intellectual disability, or similar) who participate in programs like Special Olympics show greater fitness, strength, aerobic capacity, self-esteem, confidence, social competence, and friendships compared to their peers who are not involved in such activities (Logan et al., 2019). Autistic children can also benefit from physical activity and exercise (Ceccarelli et al., 2020; Monteiro et al., 2022; Ruggeri et al., 2020), specifically experiencing positive changes in communication, social interaction, social cognition, social motivation, regulation of repetitive behaviors and awareness (Huang et al., 2020; Koh, 2024; Liang et al., 2022; Rafie et al., 2017). Physical activities like sports can also expose children to varied external stimuli, engaging multiple senses—sight, hearing, touch, and smell—and promoting sensory integration through nervous system activation and neural development (Rafie et al., 2017). Exercise further supports motor skill development, executive function, and overall quality of life for neurodivergent children (Bremer et al., 2016; St John et al., 2020; Sun et al., 2022). Collectively, this evidence underscores the profound impact of physical activity in fostering the development and wellbeing of all children, with especially significant benefits for neurodivergent children.

### Current challenges in youth physical activity participation

Despite these benefits, few children and youth are regularly physically active (Piercy and Troiano, 2018). According to the 2024 United States Report Card on Physical Activity for Children and Youth, only 20%–28% of children aged 6 to 17 meet the recommended 60 min of daily physical activity (Alliance, 2024). These data, drawn from the National Survey of Children’s Health and National Health and Nutrition Examination Survey, show even lower levels of activity among neurodivergent children, with just 19% meeting the recommended amount (Alliance, 2024; Case et al., 2020). These findings are consistent with previous research using large datasets (McCoy et al., 2016) or objective measurements (Liang et al., 2020) and highlight the urgent need for targeted interventions to increase physical activity levels, particularly among neurodivergent children.

Low motor competence plays a significant role in these low physical activity levels. Research shows that neurotypical children with motor competence below an “average” threshold are unlikely to meet recommended physical activity guidelines (De Meester et al., 2018). Additionally, many neurodivergent children exhibit fundamental motor skill differences, such as delays or challenges in balance, aiming, catching, and strength (Staples and Reid, 2010). However, findings in the neurodivergent population are mixed (Taylor et al., 2024). Some studies show only a weak association between low motor competence and physical activity levels, with motor competence varying across specific domains (e.g., upper limb coordination versus running speed). These domain-specific difficulties may influence participation in certain types of activities.

Perceived motor competence is particularly important for motivating autistic youth to engage in physical activity (Wong et al., 2024) but appears less influential for children with intellectual disabilities or ADHD. This highlights the need for programs that address not only motor skills but also the diverse psychological needs of neurodivergent children to support their engagement in physical activity.

Although person-centered factors like motor competence affect participation, features of the social and physical environment also exacerbate disparities in physical activity participation among neurodivergent children (Wong et al., 2024; King et al., 2003). One major issue is the limited understanding among instructors and coaches about neurodivergent children’s abilities. Caregivers, instructors, and physical educators may not be comfortable modifying exercise instructions for a person with high communication or cognitive support needs (McDermott et al., 2022; Parsons et al., 2024). Playground design poses another barrier, since most fail to accommodate the needs of neurodivergent children (Johnson et al., 2021). Key features such as wayfinding systems, stable surfacing, flush transitions, and sensory elements are often missing, making spontaneous physical activity less safe, engaging, and accessible. Finally, there is a notable shortage of evidence-based, free resources, including specialized curricula and community programs, to support parents in addressing their children’s physical activity needs (Rimmer and Rowland, 2008). These gaps further exacerbate the barriers to physical activity faced by neurodivergent children.

### Strategies for inclusive physical activity

Addressing these challenges requires developing targeted strategies to promote inclusive physical activity for neurodivergent children (Lakowski and Long, 2011; Murphy and Carbone, 2008). However, creating new interventions from scratch can be time-consuming and resource-intensive, making it more efficient to adapt existing evidence-based interventions (Wang et al., 2018). Adapting interventions involves modifying evidence-based interventions while maintaining their core components to ensure effectiveness (Baumann et al., 2018). This process has previously involved consideration of factors such as race, ethnicity, service setting, location, and organizational characteristics (Wiltsey Stirman et al., 2015). Yet, few researchers have applied adaptation frameworks to enhance equity and inclusion in physical activity interventions (Hasson et al., 2022; Scott-Andrews et al., 2021).

### The current project: *InPACT for Everyone*

The main objective of the current project was to systematically adapt the *Interrupting Prolonged sitting with ACTivity (InPACT) at Home* program to enable neurodivergent children to engage in physical activity at home safely. This adaptation effort, named *InPACT for Everyone*, was guided by two key principles: rapid-cycle research and the Universal Design for Learning (UDL) principle of Representation (Johnson et al., 2015; Meyer et al., 2014). Rapid-cycle research is a systematic process where research teams identify barriers and address them using practice-based evidence, facilitating a swift transition from concept development to implementation in practice (Johnson et al., 2015). We previously used this approach to adapt a classroom-based physical activity intervention for delivery in the home (Hasson et al., 2022). The UDL principle of Representation emphasizes the need to represent information in multiple ways (e.g., through differing modalities such as text, images, and audio) to maximize accessibility of instructional content for learners with diverse backgrounds and support needs (Lieberman et al., 2021; Lieberman and Houston-Wilson, 2018). Multiple means of representation should also be provided in the assessment process, so that learners have a variety of opportunities to demonstrate skills.

The current paper describes key steps in the systematic adaptation of the *InPACT at Home* program, leveraging these frameworks to increase access to health-enhancing physical activity for neurodivergent children. By enhancing flexibility while maintaining fidelity, *InPACT for Everyone* seeks to address health equity and foster inclusive environments for physical activity.

## Methods

The *InPACT at Home* program was chosen for adaptation due to its extensive reach across the state of Michigan. Specifically, when the program launched, website reach included all 83 counties and the daily viewership on public television ranged from 15,000–20,000 (Hasson et al., 2022). The *InPACT at Home* program was developed during the COVID-19 pandemic to provide equitable opportunities for physical activity in the home environment and address growing concerns of inactivity and obesity during the shelter-in-place restriction. Adapted from an existing classroom-based physical activity intervention, the *InPACT at Home* program retained key elements such as use of exercise videos, maintenance of intervention dose, and instructor-led physical activities (Hasson et al., 2021). The program features 132, 8-min exercise videos created by physical education teachers, pediatric exercise physiologists, athletes, and other fitness professionals. These videos focus on four main types of exercise: cardio, strength, sports skills, and mindfulness. The videos have been evaluated as high quality regarding their production (Beemer et al., 2023). Children have rated the program as enjoyable and can feasibly complete two videos at home (Beemer et al., 2022; Beemer et al., 2024).

### Rapid adaptation of the *InPACT at Home* program

In June 2023, an expert in neurodivergent movement (H.L.M.) reached out to the program director of the *InPACT at Home* program (R.E.H.) to discuss increasing inclusive physical activity opportunities for youth. The principal investigator agreed and formed an interdisciplinary team of researchers and practitioners with expertise in motor development, pediatric exercise physiology, inclusive physical activity practices, and adapted physical education to address this issue (see Table 1). The *InPACT* program director contacted each team member via email to confirm their involvement in the adaptation team. Approximately 55% of the team members identified as women of color (including African American, Asian, Hispanic, and Middle East and North Africa [MENA]), and the entire team was composed of women. In addition, one of the team members identified as neurodivergent and had a neurodivergent immediate family member.

**TABLE 1:** Team member expertise. The specific expertise of each member of the video review team and expert team.

Team member name	Expertise
Video reviewer 1	A research assistant with a B.S. in Biopsychology, Cognition, and Neuroscience from the University of Michigan, with experience in developmental psychology and fitness app development for neurodivergent children
Video reviewer 2	An undergraduate research assistant at the University of Michigan specializing in Biology, Health, and Society, with experience as a dance instructor
Video reviewer 3	An undergraduate research assistant at the University of Michigan specializing in Biology, Health, and Society, with experience in pediatric exercise physiology
Video reviewer 4	An undergraduate research assistant at the University of Michigan specializing in Applied Exercise Science, with practical experience in sports
Video reviewer 5	An undergraduate research assistant at the University of Michigan specializing in Movement Science, with practical experience in sports
Video coder 1	A research assistant with a B.S. in Applied Exercise Science from the University of Michigan, with experience in strength training and fitness instruction
Video coder 2	A research assistant with a B.S. in Movement Science from the University of Michigan, with experience working with kids with ASD, Down syndrome, and other processing disorders
Neurodivergent movement expert researcher (H.L.M.)	A researcher specializing in neurodivergent movement, with 12 years of experience developing and implementing physical activity interventions in pediatric populations affected by ASD.
Adapted physical education expert researcher (L.R.K.)	A researcher specializing in adapted physical education, with 15 years of experience in developing and promoting early motor and physical activity interventions for special populations
Adapted physical education practitioner (A.P.)	An experienced practitioner specializing in kinesiology with over 20 years of teaching adapted physical education in public schools
Physical education researcher and practitioner (K.W.)	An experienced researcher and practitioner specializing in physical education, with 38 years teaching Physical education and 25 years managing community-based physical activity programs for both typically developing and neurodivergent youth
Video script development expert (J.M.B)	A researcher and general educator with 11 years’ experience developing interventions that support health behaviors across communities and lifespans

This diversity played a pivotal role in shaping the adaptation process by bringing together multiple cultural and experiential perspectives. The team’s varied social backgrounds and professional expertise ensured the adapted intervention would be inclusive and accessible to a wide range of neurodivergent children. With women of color from diverse cultural and neurodivergent backgrounds on the team, the adaptations were crafted with an enhanced sensitivity to how social, cultural, and cognitive factors affect engagement, comprehension, and comfort for children from different backgrounds. This inclusivity, paired with interdisciplinary expertise, enabled the team to create a nuanced, culturally sensitive adapted intervention to meet the unique needs of neurodivergent children.

### Rapid adaptation of the *InPACT at Home* program

The six steps of rapid-cycle research provide a structured approach to developing, testing, and scaling solutions. These steps are based on the Framework for Rapid-Cycle Research developed by the Agency for Healthcare Research and Quality, which outlines the phases of the adaptation and research process from conceptualization to implementation (Johnson et al., 2015). The process begins with *preparation*, where partner organizations are identified, along with individuals within those organizations who will champion the program. This is followed by *problem exploration*, which involves understanding key problems that need to be solved. This stage emphasizes dialogue and sharing diverse perspectives to deepen the understanding of the issue. Next is *knowledge exploration*, which examines the problem from multiple angles. This includes characterizing the problem, identifying industries that face similar challenges, pinpointing organizations within those industries that have successfully addressed the problem, and analyzing the processes or activities that set these organizations apart.

*Solution development* then focuses on identifying the simplest, least invasive, and most scalable solutions that can be applied. Once potential solutions are developed, the *solution testing* phase evaluates their effectiveness. Project team members assess the importance of and progress on various evaluation dimensions (Reach, Effectiveness, Adoption, Implementation, Maintenance or RE-AIM). Based on these assessments, teams prioritize one or two RE-AIM dimensions, set proximal goals, and implement strategies to improve their progress. This step follows an iterative process to refine the solutions. Finally, the *dissemination* phase ensures the findings and effective solutions are shared, facilitating broader adoption and scaling.

This project specifically focused on the second through fourth phases: *problem exploration*, *knowledge exploration*, and *solution development*. Each of these three steps of rapid-cycle research and their associated activities are described in Table 2. The fifth and sixth phases (*solution testing* and *dissemination*) were beyond the scope of this project and the *preparation* phase was accomplished prior to the adoption of the rapid cycle framework through the formation of the adaptation team.

**TABLE 2:** Key cycle steps to adapt the *InPACT at Home* program for use in neurodivergent pediatric populations.

Rapid cycle steps	Description	Activities	Outcome
Problem exploration *Understanding key problems that need to be solved*	Identified problem: motor skill level required to complete *InPACT at Home* exercise videos was a barrier to for both neurotypical and neurodivergent children	1. Assembled a video review team and coders 2. Identified exercises in videos 3. Standardized names of exercises 4. Categorized exercises based on muscle group/motor skill	1. Video review team composed of research assistants with expertise in pediatric exercise physiology and motor development 2. 500+ unique activities identified 3. 260 standardized exercises cataloged 4. 7 motor skill categories developed
Knowledge exploration *Examining the problem from multiple perspectives*	Questions asked: • How many activities need step-by-step instructions? • What, if any, free resources are available? • How would the instructions be developed?	1. Assembled an expert team to review the list and categories of exercises 2. Expert team searched for free resources that offered step-by-step instructions for basic movements 3. Expert team provided recommendations on how to develop and refine step-by-step instructions from AI-generated output, if free resources were not available	1. Experts included practitioners and researchers 2. Expert team identified no available resources that met the needs of this project 3. Recommendations for refining instructions included: • Step-by-step instructions for all exercises • Tailoring ChatGPT prompts • Applying UDL principles • Avoiding figurative language • The Three C’s • Cognitive Load Management • Standardized Starting Positions • Encouraging Continuation
Solution development *Identifying the simplest least invasive, and most scalable solutions that can be applied*	Solution: Use Generative AI to develop prompts to create instructions with expert team refinement Expert team development of cue words and videos	1. Novice AI prompt developer recruited 2. ChatGPT used to develop prompts through an iterative process 3. Generated prompts used to develop exercise instructions and cues 4. Expert team modified the ChatGPT instructional output for content and style 5. Experts identified exercises that were “complex” 6. Instructional videos were developed	1. Novice developer with 100 h of experience using ChatGPT was selected 2. Two prompts were created 3. Review team extracted important information from the Chat-GPT instructions 4. Expert team reviewed instructions and cues for instructional content validity 5. A “burpee” was identified as a highly complex movement 6. 24, 2-min instruction videos were created to scaffold learning to complete a “burpee”

### Problem exploration-understanding the problems that were important to solve

The adaptation team identified that the difficulty level of the *InPACT at Home* exercises posed a barrier to engagement for both neurotypical and neurodivergent children. Previous research found that the skill level needed to complete the exercises was a challenge for neurotypical children (Beemer et al., 2022). This suggested that skill level may also be a barrier for neurodivergent children. To improve program engagement for both neurotypical and neurodivergent children, it became necessary to identify which exercises featured in the videos required additional instructions, cues, and demonstrations to support participation. The following sections outline the activities conducted during the *problem exploration* phase.

#### Activity 1: assemble a video review team

During the *problem exploration* phase, the adaptation team formed a video review team to identify and categorize exercises in the *InPACT at Home* videos that required additional instructions, cues, and demonstrations (see Table 1). For this project, an exercise was defined as “a series of skills or movements performed to strengthen one or more muscle groups”.

The video review team had two distinct roles: “video reviewers”, who systematically documented exercise names in the videos, and “coders”, who standardized these names by grouping similar exercises under a single term. Standardizing exercise names aimed to reduce variations in terminology and concepts, minimizing the cognitive load on the learner’s working memory (van Merrienboer and Sweller, 2010). As a result, children could focus more on performing the movements rather than processing different terms or ideas.

#### Activity 2: identify exercises in videos

Each *InPACT at Home* video was reviewed to identify the exercises included. Each reviewers watched a subsection of the video catalog, noting every exercise taught and any unique exercise names that needed standardization. This process produced a comprehensive list of movements and exercises found in all *InPACT at Home* videos.

#### Activity 3: standardize exercise names

The coders then assigned each unique exercise a more standardized name (e.g., “stand-up sit-downs” were grouped under “squats”). To accomplish this, the coders examined each uniquely named exercise, watched the corresponding video segments, and identified its key movements. If those movements matched an exercise already on the standardized list, the coders grouped the activity under the appropriate name, eliminating the unique names from the final list.

#### Activity 4: categorize standardized exercises by muscle group or motor skill

After standardizing the exercise names, exercises were categorized based on the muscle group or movement skill they primarily targeted. The team coded the standardized list of exercises from the *InPACT at Home* videos, focusing on the main body parts and skills activated during the movements. Recognizing that no exercise exclusively targets a single body part or skill, the video review team determined which body part was most activated or which skill was most integral to each movement.

### Knowledge exploration—Exploring the problem from different perspectives

The following sections outline the activities conducted during the *knowledge exploration* phase.

#### Activity 1: assemble an expert team

Drawing from insights gained during the problem exploration phase, the adaptation team brought together a group of experts—comprising researchers and practitioners from within the team—during the *knowledge exploration* phase. This team (A.P., H.L.M., K.W., and L.K.) reviewed the list and categories of exercises and provided guidance on developing step-by-step movement instructions tailored to meet the needs of neurodivergent children.

#### Activity 2: expert consultations to identify existing free resources

Members of the adaptation team (A.S., T.S., and R.E.H.) conducted a series of meetings with practitioners (A.P. and K.W.) from the expert team to explore the availability of free resources offering step-by-step instructions for fundamental movement skills. These discussions also addressed potential strategies for developing such instructions if free resources were not available. The team specifically considered the utility of generative artificial intelligence (AI) for creating tailored resources for neurodivergent children.

ChatGPT 3.5, a free AI chatbot developed by OpenAI, emerged from the discussions as a promising tool due to its ability to quickly provide accurate information and enable customization through interactive prompts (Ray, 2023). This capability allows users to tailor outputs to specific needs, making it a valuable resource for developing inclusive instructional materials. Additionally, using generative AI software would enable the adaptation team to efficiently develop and refine instructions for hundreds of activities—a task that would have otherwise taken months to complete.

In reviewing the literature, the adaptation team confirmed that generative AI had already been successfully used in fitness and medicine to instruct and inform users. For example, researchers in Finland used ChatGPT 4.0 to generate personalized workout plans that participants found to be high quality, engaging, and useful (Logacheva et al., 2014). Another study showed that medical students in Canada were more successful at learning surgical skills in simulations when tutored and given feedback by an AI tutor rather than an expert instructor (Fazlollahi et al., 2022).

While AI technologies are not yet recommended as replacements for personalized, progressive, and health condition-specific guidance from healthcare and fitness professionals, they can serve as valuable supplementary tools. In their current form, AI technologies enhance accessibility, particularly for individuals who cannot afford professional advice. Consequently, the practitioners discussed using generative AI alongside expert feedback to develop step-by-step instructions for inclusive physical activity.

#### Activity 3: expert recommendations for developing and refining instructions and cue words

If new resources needed to be developed, the expert team agreed to provide recommendations on how to develop and refine instructions and cue words from AI-generated output.

### Solution development—identifying the simplest possible solution to be applied

The following sections outline the activities conducted during the *solution development* phase.

#### Activity 1: novice AI prompt developer recruitment

To assist in developing new instructions and cue words, a novice AI prompt developer was recruited. The decision to use a novice developer instead of a professional prompt engineer was intentional, aimed at simulating the experience of an average user with limited or no experience using ChatGPT. This approach ensured that generating instructions would be feasible for parents and families.

#### Activity 2: ChatGPT prompt development

The process of developing ChatGPT prompts was iterative. Initially, the bot was asked to provide instructions for a sample exercise (lateral lunge), and the outputs were then refined to match the desired content and format. For instance, if ChatGPT produced lengthy instructions (more than 10 steps), it was prompted to condense them. If the language used was not straightforward (e.g., using metaphors or elaborate sentences to describe the step) or was too verbose, it was instructed to simplify the wording. Subsequently, ChatGPT was asked, “What prompt should I use to get this output?” The returned output would then be used as a template prompt for each exercise on the standardized list by substituting “lateral lunge” for the exercise of interest (Prompt 1).

**FIGURE 1:**
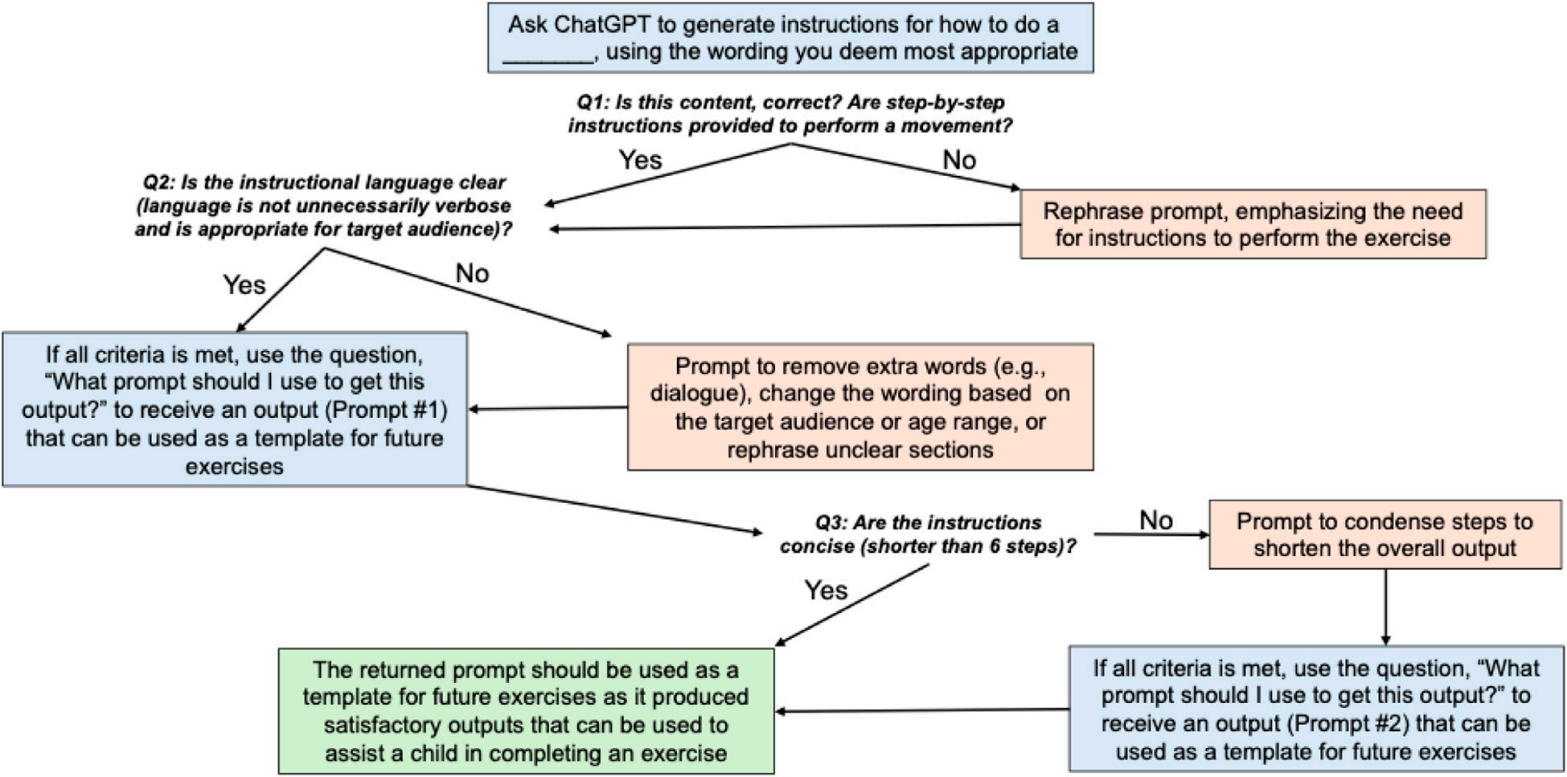
The decision tree used to initially generate ChatGPT prompts that return satisfactory exercise instructions. *Note: This study uses Prompt #1 and Prompt #2 in conjunction to generate instructions for each exercise. However, for replication purposes, users may be able to use only Prompt #1 or Prompt #2 to generate satisfactory results depending on what is returned by ChatGPT.

A similar process was used to develop a prompt that further shortened the length of the instructions, helping the content become more digestible (Prompt 2). The combination of both prompts allowed for the instructional content to be accurate (Prompt 1) and concise (Prompt 2). Figure 1 displays the series of ChatGPT inputs used in the form of a decision tree to generate prompts for creating inclusive step-by-step instructions. Supplementary Appendix S1 provides an example of the iterative process used to generate and refine exercise instructions from ChatGPT.

Step 1: Development of step-by-step instructions and cue words

After finalizing the two template ChatGPT prompts, the AI prompt developer used the prompts to generate step-by-step exercise instructions for each activity on the standardized list of exercises.

Step 2: Refinement of step-by-step instructions

Once the AI-generated exercise instructions were created, members of the expert team reviewed and approved them for both content validity and style consistency. Based on this review, a set of expert recommendations was compiled to assist parents and families in using generative AI to develop their own instructions for additional exercises beyond those included in the *InPACT at Home* program.

Step 3: Cue word development and refinement

After the expert team reviewed and approved the AI-generated instructions for content and style, the video review team began developing short cue words (2-3 words) to reinforce key concepts from the instructions. The focus was on content validity, ensuring that the cue words were derived directly from the longer instructions.

The video review team assessed the instructions to identify critical words related to physical form or specific body movements. For example, the AI-generated instructions for a squat exercise might read:

Starting Position: 1) Stand with feet hip-width apart. 2) Bend Knees: Slowly bend knees, like sitting in a chair. 3) Keep Back Straight: Maintain a straight back. 4) Go Down Comfortably: Lower body as far as comfortable. 5) Push Up: Push through heels to stand straight.

From this, the expert feedback led to refined instructions: 1) Stand with feet hip width apart; 2) Pretend you’re sitting in a chair; 3) Push through heels to stand.

The corresponding cue words, developed by the review team, would then be: 1. Feet apart; 2. Pretend chair sit; 3. Push to stand.

The expert team then verified that each cue word reinforced essential information for completing the movement. By combining AI-generated instructions with expert feedback to refine the cues, the new resources were reviewed at multiple levels—iteratively refined by AI, the video review team, and the expert team—to ensure the content was accurate and beneficial for both neurotypical and neurodivergent children.

Step 4: Expert identification of “complex” exercises

After developing the step-by-step exercise instructions and cue words, the expert team identified which exercises were “complex”. These exercises were deemed particularly challenging for children due to the involvement of multiple compound movements. For these exercises it was recommended that children would benefit from demonstration videos to better understand these movements. This aligned with the UDL principle of Representation, which emphasizes presenting information in multiple formats to support diverse ways of learning and engaging with instructional content (Meyer et al., 2014).

The expert team identified specific complex exercises in the *InPACT at Home* videos that could be broken down into individual movements and then scaffolded into a video series teaching comprehensive whole-body complex movements. Additionally, it was determined that these step-by-step instructional videos could serve as introductory videos for both neurodivergent and neurotypical youth who are new to the *InPACT at Home* program. This approach ensured that all participants, regardless of their developmental background, could effectively learn and engage with the *InPACT at Home* exercises.

Step 5: Video development

A member of the expert team, A.P., was chosen to appear in the instructional videos due to her previous experience in exercise video production. An expert in video script development (J.B.) was recruited to join the expert team to create the video content, while R.E.H., the *InPACT* program director, served as the scientific advisor to ensure the accuracy of the video content. All videos were filmed at a community location using professional equipment and staff.

## Results

### Problem exploration

#### Outcome 1: composition of the video review team

The video review team consisted of graduate and undergraduate research assistants from the adaptation team with expertise in pediatric exercise physiology and motor development. Five assistants were assigned as video reviewers, while the other two served as video coders.

#### Outcome 2: identification of unique exercises

From the 132 videos in the *InPACT at Home* program, more than 500 unique exercises were identified.

#### Outcome 3: standardization of exercises

The 500+ unique exercises were consolidated into a list of 259 standardized exercises.

#### Outcome 4: standardized exercises categorized by muscle group or motor skill

The list of standardized exercises was then coded into one of seven fundamental motor skill categories based on the skill that was performed: jumping, lateral movement, core, upper body, lower body, sports skills, and compound movement. Table 3 describes the key features and examples of each category. For example, the “squat” exercise was initially identified to target glutes and quadriceps muscles. This was then coded into the lower body category, as the muscles predominantly involved were part of lower body muscle groups. This coding process helped the team visualize the distribution of exercises across motor skills categories. From this analysis, there were 11 jumping exercises, 4 lateral movement exercises, 28 core exercises, 61 upper body exercises, 68 lower body exercises, 48 sports skill movements, and 39 compound movement exercises.

**TABLE 3:** *InPACT at Home* Exercise Categories. This table displays each category of exercise present in the *InPACT at Home* program. Each category includes distinct key features that help determine which category each exercise belongs in. The table also provides examples of exercises in each category.

Category	Key features	Example
Jumping	Jumping as the predominant movement	Jump rope, star jumps
Lateral movement	Moving side to side	Lateral foot taps, lateral line jumps
Core	Exercises that predominantly engage core muscle groups	Crunches, heel taps
Upper body	Focused movement above the torso, including arms, shoulders, and upper back	Arm circles, jabs
Lower body	Focused movement below the torso, including glutes, quads, hamstrings, and calves	Squats, butt kicks
Sports skills	Activities that simulate skills needed to play a sport	Overhand throw, soccer dribbles
Compound movement	Exercises that involve two or more major categories	Burpees, planks

### Knowledge exploration

#### Outcome 1: expert team assembled

The expert team consisted of four researchers and/or practitioners with expertise in neurodivergent movement, adapted physical education, and physical education. This team reviewed a subset of the compiled list of activities and developed a strategy for creating a glossary of exercise instructions.

#### Outcome 2: expert identification of available resources

Consultations with the practitioners on the expert team revealed that while validated adapted physical activity resources exist, many come with associated costs and are not freely accessible to the public. The free resources that are available do not include step-by-step instructions for teaching fundamental motor skills. As a result, the decision was made to use ChatGPT to generate step-by-step instructions.

#### Outcome 3: expert recommendations for developing and refining instructions from AI-generated output

Throughout the project, nine meetings with the expert team were conducted, leading to several key recommendations for developing and refining the AI-generated exercise instructions:

##### Step-by-Step instructions for all exercises

Early discussions with researchers H.L.M. and L.R.K. led to the recommendation that all exercises, regardless of complexity, should include step-by-step instructions. This approach was chosen due to the lack of a clear definition for complex exercises and the need to address individual differences, particularly for neurodivergent children.

##### Tailoring ChatGPT prompts for neurodivergent children

The team emphasized the importance of adjusting ChatGPT prompts to meet the unique needs of neurodivergent children, who often have different body awareness compared to neurotypical peers (Fears et al., 2023). The researchers highlighted that any movement is beneficial, even if it does not perfectly match the cue words and instructions.

##### Applying UDL principles

A strong focus was placed on UDL principles. The team stressed the need to include both written descriptions and visual aids (e.g., pictures and videos) to accommodate diverse learning preferences and enhancing accessibility. Accordingly, the expert team recommended presenting instructional content in three formats: detailed instructions, short cues, and videos. This variety allows children to learn in the format that best suits their needs, whether through text or visual aids, and helps ensure accessibility for all learners (Meyer et al., 2014).

##### Avoiding figurative language

Practitioners A.P. and K.W. advised against using figurative language (e.g., metaphors like “keep your eyes on the target”) in the instructions (Holt and Ratliffe, 2004), as neurodivergent children often struggle with figurative language. Instead, they recommended clear, straightforward language to improve understanding (Kalandadze et al., 2018).

##### The three C’s

The expert team recommended focusing on the 3 C’s for instructional content—conciseness, clarity, and consistency—to reduce cognitive load and improve comprehension (Sweller et al., 2011). Conciseness refers to using as few words as possible, clarity involves using simple and unambiguous language, and consistency requires using the same terminology for each activity.

##### Cognitive load management

The team recommended limiting each exercise to three-to-five cues, with each cue being two-to-three words long. This helps prevent cognitive overload, as research indicates that working memory typically holds only three-to-four pieces of information (Farrington, 2011).

##### Standardized starting positions

To reduce unnecessary wording, the expert team established four standardized starting position names: “ready position” (stand upright, feet together), “stand comfortably” (stand upright, feet shoulder-width apart), “lie down” (lie on back with body parts flat on the ground), and “bend knees” (feet shoulder-width apart, in a half-squat position).

##### Encouraging continuation

The expert team also recommended adding the phrase “Keep Going!” at the end of instructions to signal that the movement should be repeated until the child feels comfortable.

### Solution development

#### Outcome 1: novice AI prompt developer identified

A member of the adaptation team with approximately 100 h of experience using ChatGPT 3.5 was selected to create concise instructions and supplementary cue words for each exercise.

#### Outcome 2: ChatGPT prompts

Through the iterative process of developing ChatGPT prompts, the team identified two complementary prompts that, when used together, offered consistent and effective instructions. These two prompts were, “Provide simplified step-by-step instructions for a [insert exercise name here] suitable for a neurodivergent child” and “Condense the step-by-step instructions for a [insert exercise name here] suitable for a neurodivergent child.”

#### Outcome 3: step-by-step instruction and cue word development

These AI-generated prompts were inserted into ChatGPT one after the other, substituting the blank for an exercise name, to result in a set of instructions to perform the movement. The review team then extracted the important information from the ChatGPT-generated instructions to develop cue words. Table 4 displays a sample of the developed instructions and cue words.

**TABLE 4:** A sample of exercises, the ChatGPT outputs, the corresponding instructions, and their cues. Short instructions and cue words were developed by the review team with expert input.

Exercise name	Category	ChatGPT output	Short instructions	Cue words
Star jumps	Jumping	1. Stand straight, arms down 2. Jump out wide, arms and legs out 3. Jump in, arms and legs down 4. Repeat, breathing steadily 5. Enjoy!	1. Stand at the ready position 2. Jump out, stretching your arms and legs out wide like a star 3. Jump in, bringing your arms back down and legs together 4. Keep going!	1. Ready position 2. Jump out 3. Jump in 4. Keep going!
Heel taps	Core	1. Lie on back with knees bent 2. Lift shoulders slightly 3. Tap right heel with right hand 4. Tap left heel with left hand 5. Repeat 6. Breathe steady 7. Have fun!	1. Lie down, with your back touching the floor 2. Bend your knees, with your feet flat on the floor 3. Squeeze your stomach muscles 4. Reach your right hand to tap your right heel 5. Reach your left hand to tap your left heel 6. Keep going, alternating your taps!	1. Lie down 2. Tap heel 3. Tap other heel 4. Keep going!
Lateral line jump	Lateral	1. Stand with feet together 2. Jump sideways 3. Jump back 4. Repeat at your own pace 5. Breathe steady 6. Have fun!	1. Stand at the ready position 2. Jump to one side 3. Jump back 3. Keep going!	1. Ready position 2. Jump sideways 3. Jump back 4. Keep going!
Small arm circles	Upper body	1. Stand with feet apart 2. Raise arms to sides 3. Make small circles forward 4. Repeat 5. Breathe steady 6. Have fun!	1. Stand at the ready position 2. Raise arms out like a ‘T’ 3. Make small circles with your arms, like stirring the air 4. Change directions, stirring the air the other way 5. Keep going!	1. Stand comfortably 2. Make a ‘T’ 2. Small circles 3. Change directions 4. Keep going!
Butt kicks	Lower body	1. Stand with feet apart 2. Lift heels towards buttocks 3. Switch legs 4. Repeat 5. Breathe steady 6. Have fun!	1. Stand at the ready position 2. Bring one heel to your butt 3. Bring the other heel to your butt 4. Hop faster from foot to foot 5. Keep going!	1. Stand comfortably 2. Heel to butt 3. Other heel 4. Faster 5. Keep going!
Overhand throw	Sport	1. Stand with feet apart 2. Hold the ball 3. Step forward with opposite foot 4. Raise arm back 5. Throw the ball forward 6. Follow through with arm 7. Repeat 8. Breathe steady 9. Have fun!	1. Find something to throw (ball, rolled socks, etc.) 2. Stand at the ready position 3. Hold item in hand 4. Raise arm, bending at elbow to make an ‘L’ shape 5. Step forward with opposite foot (right hand, left foot) 6. Twist upper body towards ‘L’ shape side 7. Throw item	1. Hold item 2. Make an ‘L’ 3. Step and twist 4. Throw

#### Outcome 4: step-by-step instruction and cue word refinement

After the initial development of cues and instructions, the expert team reviewed them for instructional content validity. They ensured that the wording for starting positions, signals for repetitions, and instructions for general movements remained consistent across each set of instructions and thoroughly covered all the necessary information to correctly perform the movement. This qualitative process helped to ensure the accuracy of the instructions and cues.

#### Outcome 5: expert identification of “complex” exercises

The burpee was identified as a highly complex movement due to its combination of multiple motor skills (e.g., jumping and balancing), compound movements (e.g., engaging the core, lower body, and jumping), and the need for precise coordination across nearly every muscle group. Given its complexity, the burpee was selected as an example for a demonstration video to guide participants in mastering the movement effectively.

#### Outcome 6: video development

**FIGURE 2:**
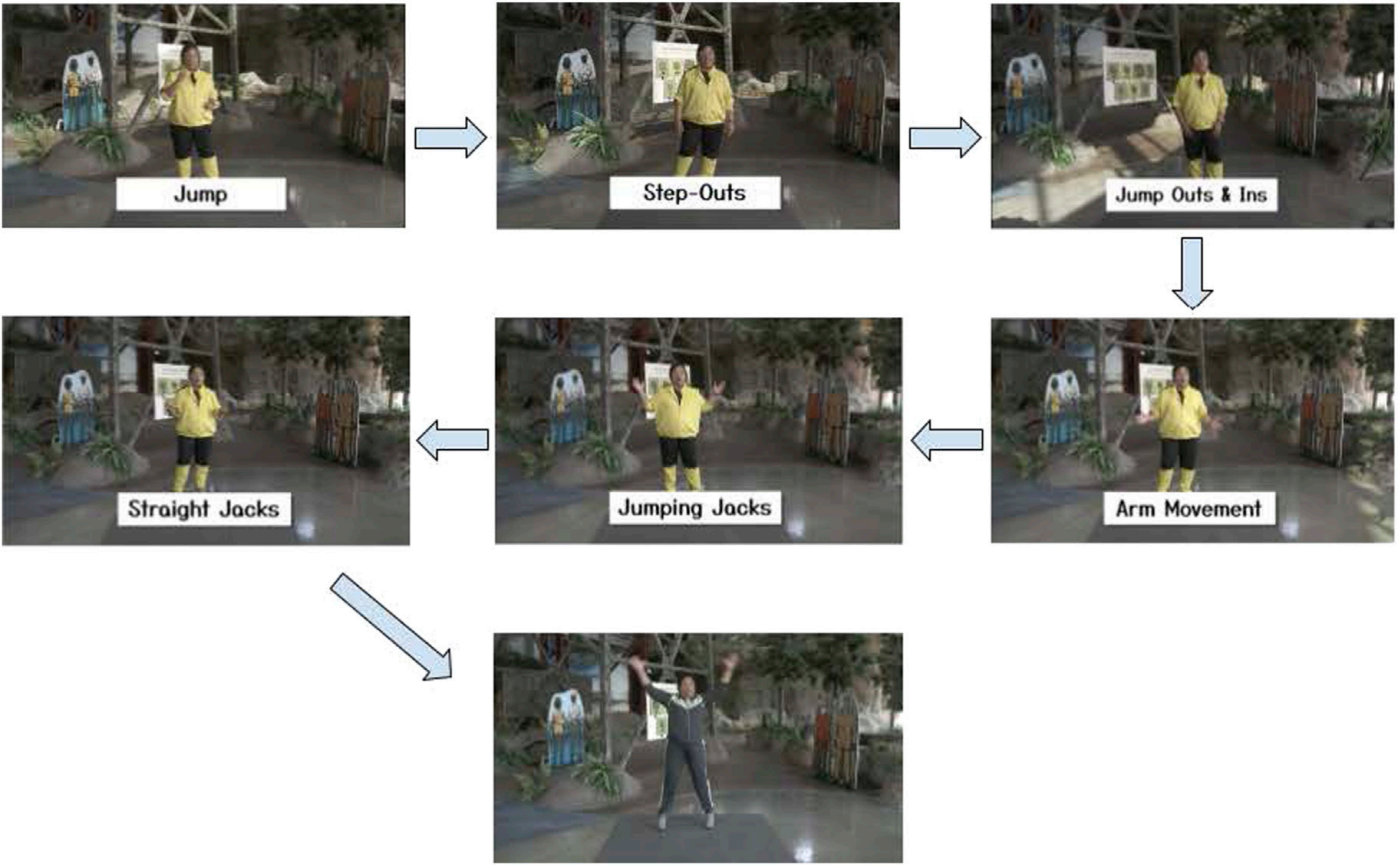
Screenshots from each of the videos in unit 1, leading up to straight jacks (a scaled-up version of jumping jacks). The last image shows the basic foundational exercise from unit 1, jumping jacks, serving as the warmup for unit 2 videos.

Figure 2 shows a visual representation of several videos designed to teach children how to perform complex exercises by breaking the movements down into manageable steps. A total of 24 videos, each 2 minutes long, was created for this purpose. These videos were organized into four units, with each unit named after the specific complex exercise covered in its series of six videos. Within every unit, exercises were ordered so that each new skill could be scaffolded onto the next, ensuring that the fundamental skills needed to complete the complex exercise were mastered before moving on to the next component. For example, completing unit 1 meant that the child would be able to perform a straight arm jack, a complex version of a jumping jack. To do this, they are taught how to jump in video 1, how to move their legs side to side in video 2, how to jump in and out in video 3, how to move their arms in video 4, how to put everything together to perform a jumping jack in video 5, and then finally how to perform a straight jack in video 6.

Each of the first three units focused on teaching different complex exercises that were about the same difficulty. Because the expert team determined that one of the most complex exercises present in the *InPACT at Home* videos was the burpee, exercises in units 1-3 focused on skills that would help perform a burpee in unit 4. These foundational skills were reinforced through each unit by making moves from the previous unit serve as the warm-up in the following unit (e.g., jumping jacks from unit 1 were the warm-up for exercises in unit 2).

To ensure equitable access, the videos are broadcast on the Michigan Learning Channel as part of their “Read, Write, ROAR!” program (https://www.michiganlearning.org). They are now also a complementary component of the *InPACT at Home* program (inpactathome.umich.edu). The instructions for all these exercises are also being compiled into a glossary and will be made available to families on the program website.

## Discussion

### Systematic adaptation process

To enhance the accessibility of the *InPACT at Home* program for neurodivergent children, a systematic adaptation approach was utilized. The process began with a video review team identifying and categorizing exercises by muscle group or skill (*problem exploration*). Following this, a series of meetings with an expert team established the criteria for creating step-by-step instructions (*knowledge exploration*). Generative AI was then used to generate initial instructions, which were reviewed, refined, and supplemented with short cues by the expert team. For exercises that were particularly complex, additional instructional videos were produced to offer further guidance (*solution development*).

This approach addressed a fundamental shortcoming of many at-home physical activity programs, which often lack options for children with diverse cognitive needs and may require financial resources or prior exercise knowledge (Rimmer and Rowland, 2008). By integrating generative AI with expert feedback, this adaptation process rapidly provided step-by-step instructions, ensuring that neurodivergent children have equitable opportunities to participate in physical activity at home. *InPACT for Everyone* includes prompts and guidelines that parents can use to generate instructions, sample instructions and cues for activities, and videos to further support participation. The outcome of this systematic process of adaptation provides families with the tools to support their children in completing virtual exercise routines without financial constraints or access barriers.

### Collaborative expertise and adaptation

Similar to the initial adaptation of the *InPACT* program from the classroom to the home environment, the *InPACT at Home* program’s adaptation relied on collaboration with researchers and practitioners specializing in motor development, pediatric exercise physiology, inclusive physical activity practices, and adapted physical education. These experts were considered “physically educated” (Decorby et al., 2005), and contributed essential expertise to teach basic movement skills, apply UDL principles, and model a range of inclusive physical activities that promote lifelong participation in physical activity.

### Role of generative AI in adaptation

In this study, ChatGPT was used to create step-by-step instructions for physical activities, leveraging a free and accessible platform to support neurodivergent children. While several programs offer evidence-based resources, their cost limits accessibility for many families. For example, Sports, Play, and Active Recreation for Kids (SPARK) is an evidence-based program that offers physical education resources and curricula to promote physical activity and healthy lifestyles in schools (McKenzie et al., 2016). SPARK is particularly useful as it breaks exercises down into manageable steps and includes inclusive physical education options. Similarly, the Effective Physical Education Curriculum (EPEC) program aims to create inclusive physical education environments and ensure accessibility for all children, including those with disabilities, by providing progressive exercises to reinforce new skills and apply them in various contexts (Michigan's Exemplary Physical Education Curriculum Project, 2000). However, both SPARK and EPEC are behind paywalls, limiting their accessibility for parents who want to guide their children in exercises.

OPEN (https://openphysed.org) provides high quality free curriculum resources to schools but this curriculum lacks specific instructor prompts for teaching fundamental motor skills. The National Center on Health, Physical Activity and Disability (https://www.nchpad.org) offers free information and exercise videos for people with physical, sensory, and cognitive disabilities, but provides limited guidance on teaching motor skills and complex movements to neurodivergent children who need additional support. Finally, resources like Steps to Inclusion (Tristani et al., 2019) provide theoretical guidance on making physical activity inclusive but can be challenging for parents without practical experience. By utilizing ChatGPT and integrating expert feedback, the current study demonstrated how AI can bridge these gaps, offering accessible and practical tools for parents and families.

### Observations on AI output and inclusivity

An interesting observation made during the project was ChatGPT’s tendency to include motivational language when prompted to develop tailored instructions for neurodivergent children. This extra content was not consistently included when ChatGPT was asked to provide instructions alone or when the target audience was specified as “kids”. Although this motivational content was removed from the instructional materials for this project due to its lack of direct relevance to the movements, it is noteworthy that ChatGPT tends to add extra information based on assumptions about the needs of neurodivergent children.

That phenomenon highlights the importance of training AI models with diverse, inclusive datasets, as an AI model’s effectiveness depends on the training data it was developed with. Research on developing inclusive AI systems highlights that the problem-solving frameworks used for training often represent a single viewpoint, which can lead to homogenization and perpetuate stereotypes or biases (Hutson, 2024). For example, the above-mentioned scenario with ChatGPT implies that neurotypical children do not require additional motivation to complete exercises, whereas neurodivergent children do. Motivation levels vary among all individuals, regardless of whether they are neurodivergent or neurotypical. Thus, a neurotypical child who needs extra motivational content might not receive it, while a neurodivergent child with strong intrinsic motivation might receive unnecessary motivational content that could detract from the specific exercise. Hutson suggests that AI models like ChatGPT should be trained with broader, more diverse data sets, including those reflecting the viewpoints of neurodivergent individuals, to enhance inclusivity and expand the model’s utility in diverse contexts (Hutson, 2024). Future research should explore strategies for optimizing AI training to better reflect the nuanced needs of all users, ensuring equitable and accurate outputs.

### UDL framework

UDL, a framework developed by the Center for Applied Special Technology (CAST), aims to enhance and optimize teaching and learning for everyone, drawing on scientific insights into how humans learn (CAST, 2024). UDL has been successfully applied in various contexts to enhance instructional content accessibility. For instance, UDL, combined with the inclusion spectrum, has helped general physical education teachers align learning goals with activities that enable neurodivergent children to interact with their peers while staying physically active (Grenier et al., 2017). Additionally, UDL, together with the Planning for All Learners (PAL) procedures, has improved the accessibility of high school reading comprehension programs (Meo, 2008). The PAL process provides a practical four-step approach for implementing UDL: (1) setting goals, (2) analyzing the current status of the curriculum and classroom, (3) applying the UDL framework to lesson and unit development, and (4) teaching these UDL-aligned lessons and units (Israel et al., 2014).

As generative AI becomes increasingly popular, more programs are beginning to combine AI with UDL to enhance inclusivity. For example, one initiative used AI to optimize UDL implementation in an online graduate course, enabling personalized learning and customized assessments and course content (Morgan et al., 2024). Another project employed AI to develop K-12 health education lesson plans incorporating UDL principles, allowing teachers with no prior experience in health education to effectively teach the content (Conrad and Rees, 2024). While generative AI has been used in education to enhance UDL applications, its combination with UDL principles for creating inclusive physical activity resources at home remains underexplored. This project addresses that gap by providing a model for integrating UDL and AI to enhance accessibility and inclusivity.

### Strengths and limitations

This study highlights several key strengths. First, it utilized implementation and dissemination science frameworks along with universal design to systematically adapt the *InPACT at Home* program. The involvement of a diverse adaptation team was another major strength, ensuring that multiple perspectives were integrated into the resource development process, thereby enhancing the accessibility of the resources for the pediatric population in Michigan. Additionally, the innovative use of ChatGPT to create inclusive instructional content was validated by incorporating expert feedback, which further strengthened the content.

However, the study also has some limitations. Previous research has highlighted AI’s tendency to “confabulate” (i.e., to make up something without malicious intent), which may have affected the accuracy of ChatGPT’s output (Alkaissi and McFarlane, 2023). That limitation was mitigated by refining the AI-generated content through expert team feedback. Another limitation is that the effectiveness of the *InPACT at Home* adaptation—specifically its step-by-step instructions, cue words, and demonstration videos—in improving program participation among neurodivergent children has not yet been rigorously tested. Additionally, the current study focuses solely on children, creating a gap in research on how these adaptations might apply to other age groups. Furthermore, the adaptation focused exclusively on the UDL principle of “Representation.” Future research should explore how the UDL principles of “Engagement” and “Action and Expression” could be employed to further optimize inclusion within the *InPACT at Home* program. Finally, since the goal of *InPACT for Everyone* is to make the program accessible to all children, further testing is needed to determine whether this approach is effective for individuals with physical or cognitive disabilities beyond neurodivergence. It is our hope that the flexibility of this approach can be adapted to benefit a broader and more diverse community.

## Conclusions

In conclusion, this study provides valuable insights into the rapid adaptation of the *InPACT at Home* program for neurodivergent children by integrating generative AI with expert feedback and grounding the process in dissemination and implementation science frameworks. That systematic approach not only addressed barriers to accessibility that often limit participation in at-home physical activity programs but also offers a replicable model for families to create their own physical activity resources.

By following specific guidelines—such as using ChatGPT prompts to generate detailed instructions, ensuring the content is concise, clear, and consistent, and presenting the instructions in various forms—families can effectively support their children’s physical activity at home. While the study highlights promising outcomes, further research is needed to rigorously test the effectiveness of these adaptations and explore additional UDL principles to enhance inclusivity. Ultimately, this work sets a precedent for promoting health equity through the systematic and inclusive design of physical activity interventions, helping to reduce physical activity disparities and foster healthy habits among *all* children.

## Data Availability

The original contributions presented in the study are included in the article/Supplementary Material, further inquiries can be directed to the corresponding author.
