# Supplementary material for: Enhancing home-based physical activity for neurodivergent children: adapting the InPACT at Home program with AI and universal design
Source: Front Physiol. 2025 Jan 7;15:1496114. doi: 10.3389/fphys.2024.1496114 (PMC11747215; doi:10.3389/fphys.2024.1496114)
Supplement: Supplementary file 1 [file Table1.docx]

**Appendix I:** The ChatGPT logs leading to the development of the final prompts.

| **Prompt** | **Rationale** | **Output from ChatGPT** |
| --- | --- | --- |
| “Can you write step-by-step instructions to teach a neurodivergent child how to do the exercise ‘lateral lunge’” | The original prompt: emphasizes the target audience (a neurodivergent child) and a sample exercise (lateral lunge) | - Certainly! Teaching a neurodivergent child how to perform a lateral lunge requires a patient and adaptable approach. Here's a step-by-step guide:   - 1. Establish a Comfortable Environment: Choose a quiet and comfortable space, free from distractions or sensory triggers. Ensure proper lighting and ventilation for a calming atmosphere.   - 2. Build Trust and Rapport: Start by establishing a positive connection with the child. Use their preferred communication style to build trust and create a supportive environment.   - 3. Demonstrate the Lateral Lunge: Model the correct lateral lunge technique in a slow and controlled manner. Utilize visual aids, such as pictures or videos, to enhance understanding.   - 4. Break Down the Lateral Lunge into Steps: Divide the lateral lunge into smaller, manageable steps. Emphasize the importance of proper form, balance, and coordination.   - 5. Use Clear and Simple Language: Communicate the instructions using straightforward and concrete language. Repeat the steps as needed, considering the child's communication preferences.   - 6. Provide Visual Cues: Use visual cues like diagrams or hand gestures to reinforce each step. Create a visual schedule or checklist to help the child follow the process.   - 7. Offer Physical Guidance: Provide gentle physical guidance to assist the child through the motion. Adjust the level of physical assistance based on the child's comfort and responsiveness.   - 8. Incorporate Sensory Input: Consider the child's sensory needs and preferences. Use sensory-friendly equipment or modifications, such as a softer surface, to enhance the experience.   - 9. Celebrate Progress: Acknowledge and celebrate each successful attempt, focusing on effort rather than perfection. Utilize positive reinforcement, such as praise or small rewards, to motivate the child.   - 10. Be Patient and Flexible: Allow the child to progress at their own pace. Modify your approach based on the child's feedback and comfort level.   - 11. Encourage Repetition and Practice: Facilitate regular practice sessions to reinforce the lateral lunge skill. Integrate the lateral lunge into daily routines to enhance muscle memory.   - 12. Seek Professional Guidance: Consult with occupational therapists or other specialists for additional strategies and support. Remember to tailor these instructions to the specific needs and preferences of the individual child. Regular communication with caregivers and professionals will ensure a collaborative and effective approach to teaching new skills. |
| “Can you come up with instructions to teach a child how to do a lateral lunge” | Rewording prompt because output related to pedagogy rather than performing the movement. ‘Neurodivergent’ was removed from the prompt to experiment with what an output without the term would look like. | - Certainly! Here are step-by-step instructions to teach a child how to do a lateral lunge:   - 1. Introduction: Start by explaining that you will be learning a fun exercise called the lateral lunge. Share that lateral lunges help improve leg strength, flexibility, and balance.   - 2. Warm-Up: Begin with a light warm-up to prepare the body for movement. Include activities like gentle jogging in place or jumping jacks.   - 3. Proper Stance: Stand with feet hip-width apart and arms relaxed at the sides. Ensure that the child is comfortable and balanced before starting.   - 4. Demonstrate: Show the child how to perform a lateral lunge by taking a step to the side with one foot while keeping the other foot stationary. As you step to the side, bend the knee of the stepping leg while keeping the other leg straight.   - 5. Break It Down: Break the lateral lunge into two simple steps: stepping to the side and bending the knee. Emphasize the importance of keeping the back straight and the chest up during the movement.   - 6. Practice the Movement: Practice the lateral lunge without going too deep initially. Encourage the child to focus on controlled movements and maintaining balance.   - 7. Repeat on Both Sides: Perform lateral lunges on both the left and right sides to ensure balanced muscle engagement. Alternate between sides to create a rhythmic flow.   - 8. Use Visual Cues: Place markers or use visual cues on the floor to guide the child's steps. This can help with spatial awareness and proper foot placement.   - 9. Encourage Arm Movement: Demonstrate how swinging the arms naturally with the movement can aid balance. Encourage the child to swing their arms in coordination with their steps.   - 10. Gradually Increase Depth: As the child becomes more comfortable, encourage them to gradually lunge deeper into the movement. Remind them to maintain good posture and control throughout.   - 11. Practice Regularly: Incorporate lateral lunges into regular exercise routines. Make it a fun activity by adding music or turning it into a game.   - 12. Provide Positive Reinforcement: Celebrate the child's efforts and progress. Offer praise for maintaining good form and completing the lateral lunges.   - 13. Cool Down: End the session with a cool-down, including gentle stretches for the legs. Reinforce the importance of cooling down to prevent muscle soreness.   - Remember to adjust the instructions based on the child's individual needs, and encourage a positive and supportive learning environment. If the child has any physical limitations or concerns, it's advisable to consult with a healthcare professional before starting a new exercise routine. |
| “How do you do a lateral lunge for neurodivergent kids” | Rewording the prompt—although there was a little more focus on content, there was still too much information about pedagogy. ‘Neurodivergent’ was added in again to focus the content back to neurodivergent children. | - Teaching a lateral lunge to neurodivergent children may require additional considerations for sensory preferences, communication styles, and potential challenges. Here's a modified approach:   - 1. Establish a Comfortable Environment: Choose a quiet and familiar space with minimal sensory distractions. Provide sensory-friendly equipment if needed, such as a soft mat or cushion.   - 2. Tailor the Introduction: Introduce the lateral lunge in a way that aligns with the child's interests. Consider incorporating visuals or verbal cues that resonate with the child's communication style.   - 3. Use Visual Supports: Provide visual aids, such as a visual schedule, pictures, or a social story, to explain the steps of the lateral lunge. Use clear and simple visuals to enhance understanding.   - 4. Break Down the Steps: Divide the lateral lunge into smaller, manageable steps. Allow the child to practice each step individually before combining them into the complete movement.   - 5. Be Mindful of Sensory Input: Consider sensory sensitivities when selecting the environment and equipment. Offer options for sensory input, such as providing fidget tools or allowing the child to choose their preferred space.   - 6. Incorporate a Preferred Mode of Communication: Use the child's preferred mode of communication, whether it's verbal, visual, or a combination of both. Be patient and attentive to the child's cues, allowing them to express themselves in their preferred way.   - 7. Provide Physical Support: Offer gentle physical guidance or support based on the child's comfort level. Adjust the level of assistance according to the child's needs and preferences.   - 8. Encourage Sensory-Friendly Modifications: Allow for modifications that accommodate sensory needs, such as using a softer surface or adjusting the depth of the lunge. Consider incorporating sensory-friendly elements, like dimmed lighting or soothing music.   - 9. Create a Predictable Routine: Establish a predictable routine for the lateral lunge, incorporating it into a familiar sequence of activities. This can help create a sense of structure and comfort for the child.   - 10. Celebrate Success and Effort: Provide positive reinforcement for every effort and successful attempt. Celebrate progress and acknowledge the child's achievements, focusing on their individual milestones.   - 11. Make it Fun: Infuse elements of play or turn the activity into a game to make it enjoyable. Use the child's interests to make the lateral lunge more engaging and motivating.   - 12. Offer Choices: Provide choices when possible, allowing the child to have a say in the pace or style of the lateral lunge. Empower them to make decisions that contribute to their sense of control. Always remain flexible and responsive to the child's needs, adjusting your approach as necessary. Consistency, patience, and individualized support are key factors in successfully teaching a lateral lunge to neurodivergent children. |
| “Break down the steps of a lateral lunge for neurodivergent kids” | Rewording prompt because output related to pedagogy rather than performing the movement | - Certainly! Breaking down the steps of a lateral lunge for neurodivergent kids involves providing clear and simple instructions, incorporating visual supports, and being mindful of individual needs. Here's a step-by-step breakdown:   - 1. Introduction: Begin by introducing the lateral lunge in a way that captures the child's interest. Use language or visuals that resonate with their preferences.   - 2. Starting Position: Stand with feet shoulder-width apart and arms relaxed at the sides. Use visual cues or verbal instructions to emphasize the starting position.   - 3. Step to the Side: Take a step to the side with one foot while keeping the other foot planted. Use clear visual markers or guiding cues on the floor to indicate the direction of the step.   - 4. Bend the Knee: As you step to the side, bend the knee of the stepping leg while keeping the other leg straight. Reinforce the idea of bending the knee slowly and in a controlled manner.   - 5. Maintain Good Posture: Emphasize keeping the back straight and the chest up throughout the movement. Use visual aids, such as pictures or a mirror, to reinforce proper posture.   - 6. Return to Starting Position: Push off the stepping foot and return to the starting position. Encourage a slow and controlled return, focusing on maintaining balance.   - 7. Repeat on the Other Side: Perform the lateral lunge on the other side by stepping to the opposite direction. Reinforce the idea of symmetry and balance by alternating between sides.   - 8. Control the Movement: Stress the importance of controlled movements and avoiding sudden or jerky motions. Use visuals or verbal cues to convey the concept of controlled, deliberate actions.   - 9. Gradually Increase Depth: As the child becomes more comfortable, encourage them to gradually lunge deeper into the movement. Allow them to progress at their own pace, ensuring they feel in control.   - 10. Practice Regularly: Incorporate regular practice sessions into the child's routine. Make it enjoyable by turning it into a game or incorporating music.   - 11. Provide Positive Reinforcement: Celebrate the child's efforts and progress. Use verbal praise, high-fives, or other preferred forms of positive reinforcement.   - 12. Cool Down: End the activity with a cool-down, incorporating gentle stretches for the legs. Reinforce the importance of cooling down to prevent muscle soreness.   - Adapt these steps based on the child's sensory preferences, communication style, and individual needs. Use a combination of visuals, clear language, and positive reinforcement to create an inclusive and supportive learning experience for neurodivergent children. |
| “Pretend like I am a neurodivergent child, explain how to do a lateral lunge” | Rewording the prompt—although there was a little more focus on content, there was still too much information about pedagogy | - Absolutely! Let's go through the steps of a lateral lunge together, imagining you are a neurodivergent child:   - 1. Hello! Today, we're going to learn a cool exercise called the lateral lunge. It's like taking a big step to the side, and it's super fun!   - 2. Stand with your feet about shoulder-width apart. Feel the ground beneath you; make sure you're comfortable and steady.   - 3. Now, take a big step to the side with one foot. Imagine you are stepping over a line or following a special path on the floor.   - 4. As you step, bend your knee on the side you stepped with, like you're sitting down a little. Keep the other leg straight and strong.   - 5. Check how your back is feeling; try to keep it straight and your chest up. This helps you stay balanced and strong.   - 6. Now, push off the foot you stepped with, and slowly come back to where you started. It's like taking a big step back.   - 7. Great job! Now, let's try the other side. Take a big step with your other foot, and remember to bend your knee and keep your back straight.   - 8. You're doing fantastic! Each time you do a lateral lunge, make sure it's a smooth and controlled movement.   - 9. If you feel comfortable, you can try going a bit deeper into the lunge as you get used to it. But remember, it's okay to go at your own pace.   - 10. Let's practice together! Step to the side, bend your knee, and then come back. Now, let's try the other side.   - 11. You're a lateral lunge pro! Remember, you can do this whenever you want. Maybe we can even make it a game or do it to your favorite music!   - 12. Awesome job! Now, let's take a moment to cool down. We can do some gentle stretches for our legs to make them feel relaxed.   - Great work today! You did an amazing job with the lateral lunge. Remember, it's all about having fun and feeling good while moving your body. Keep it up! |
| “Can you cut out some of the dialogue” | Very lengthy output, too much of a conversational tone | - Absolutely! Let's simplify the instructions for the lateral lunge:   - 1. Hi there! Today, we'll learn the lateral lunge—a fun exercise.   - 2. Stand with feet shoulder-width apart. Get comfy and feel steady.   - 3. Take a big step to the side with one foot, like following a special path.   - 4. Bend the knee on the side you stepped with, like sitting down a little. Keep the other leg straight and strong.   - 5. Keep your back straight and chest up for balance.   - 6. Push off the foot you stepped with, slowly coming back to where you started.   - 7. Now, let's try the other side—step, bend, and come back.   - 8. Remember, smooth and controlled movements are key.   - 9. If you feel good, try going a bit deeper into the lunge, but go at your own pace.   - 10. Practice together—step, bend, and return. Try the other side.   - 11. Great job! Make it a game or do it to your favorite music!   - 12. Awesome work! Let's cool down with gentle leg stretches. Keep it up and have fun! |
| What prompt should I use to get this output | Each step was shorter and the instructions were more clear. Wanted to keep these elements for other exercises | - To get the simplified instructions for the lateral lunge, you can use a prompt like: - **"Provide simplified step-by-step instructions for a lateral lunge suitable for a neurodivergent child."** |
| “Provide simplified step-by-step instructions for a lateral lunge suitable for a neurodivergent child.” | Testing out new prompt to ensure important elements retained | - Certainly! Here are simplified step-by-step instructions for a lateral lunge suitable for a neurodivergent child:   - 1. Hello! Let's learn a fun exercise called the lateral lunge.   - 2. Stand with your feet shoulder-width apart, feeling comfortable and steady.   - 3. Take a big step to the side with one foot, like you're following a special path.   - 4. Bend the knee on the side you stepped with, like you're sitting down a bit. Keep the other leg straight and strong.   - 5. Keep your back straight and chest up for balance.   - 6. Push off the foot you stepped with, slowly coming back to where you started.   - 7. Now, let's try the other side—step, bend, and come back.   - 8. Remember, go smoothly and control your movements.   - 9. If you feel good, try going a bit deeper into the lunge, but go at your own pace.   - 10. Practice together—step, bend, and return. Try the other side.   - 11. Great job! Make it a game or do it to your favorite music!   - 12. Awesome work! Let's cool down with gentle leg stretches. Keep it up and have fun! |
| “Can you condense some of these steps” | Length and content of steps was satisfactory, but output and number of steps was still too long. Output needed to be reduced to key features only | - Certainly! Here's a condensed version of the lateral lunge instructions:   - Introduction:     - Let's try a fun exercise called the lateral lunge!   - Starting Position:     - Stand comfortably with feet shoulder-width apart.   - Side Step:     - Take a big step to the side, following a special path.   - Bend and Balance:     - Bend the knee on the side you stepped with, keeping the other leg straight.     - Keep your back straight and chest up for balance.   - Return:     - Push off the stepping foot and return to the starting position.   - Try the Other Side:     - Repeat the steps for the other side.   - Smooth Movements:     - Keep your movements smooth and controlled.   - Deeper Lunge (Optional):     - If you feel comfortable, try going a bit deeper into the lunge.   - Practice:     - Practice stepping, bending, and returning on both sides.   - Add Fun:     - Make it a game or do it to your favorite music!   - Cool Down:     - Finish with gentle leg stretches.   - Great Job! Keep it up and have fun! |
| “What prompt should I use to get this output” | Satisfied with amount of steps (6 steps involving key instructions to perform the movement itself) | - To get the condensed version of the lateral lunge instructions, you can use the following prompt: **"Condense the step-by-step instructions for a lateral lunge suitable for a neurodivergent child."** |
| “Condense the step-by-step instructions for a lateral lunge suitable for a neurodivergent child” | Testing out new prompt to ensure important elements retained | - Stand tall with feet together. - Step to the right. - Bend right knee, keep left leg straight. - Keep chest up. - Push back to starting position. - Step to the left and repeat. - Alternate sides. - Have fun! |
